# Developing evidence-based guidance for assessment of suspected infections in care home residents

**DOI:** 10.1186/s12877-020-1467-6

**Published:** 2020-02-14

**Authors:** Carmel Hughes, David R. Ellard, Anne Campbell, Rachel Potter, Catherine Shaw, Evie Gardner, Ashley Agus, Dermot O’Reilly, Martin Underwood, Mark Loeb, Bob Stafford, Michael Tunney

**Affiliations:** 1 School of Pharmacy, Queen’s University Belfast, Belfast, UK; 2 Warwick Clinical Trials Unit, The University of Warwick, Coventry, UK; 3 University Hospitals of Coventry and Warwickshire, Coventry, UK; 4 Faculty of Medicine, Department of Infectious Diseases, Imperial College London, London, UK; 5 Northern Ireland Clinical Trials Unit, The Royal Hospitals, Belfast, UK; 6 Centre for Public Health, Queen’s University Belfast, Belfast, UK; 7 Department of Pathology and Molecular Medicine, McMaster University, Hamilton, Canada; 8 Orchard Care Homes, Preston, UK

**Keywords:** Infections, Prescribing, Care homes, Older people, Algorithm

## Abstract

**Background:**

The aim of this study was to update and refine an algorithm, originally developed in Canada, to assist care home staff to manage residents with suspected infection in the United Kingdom care home setting. The infections of interest were urinary tract infections, respiratory tract infections and skin and soft tissue infection.

**Method:**

We used a multi-faceted process involving a literature review, consensus meeting [nominal group technique involving general practitioners (GPs) and specialists in geriatric medicine and clinical microbiology], focus groups (care home staff and resident family members) and interviews (GPs), alongside continual iterative internal review and analysis within the research team.

**Results:**

Six publications were identified in the literature which met inclusion criteria. These were used to update the algorithm which was presented to a consensus meeting (four participants all with a medical background) which discussed and agreed to inclusion of signs and symptoms, and the algorithm format. Focus groups and interview participants could see the value in the algorithm, and staff often reported that it reflected their usual practice. There were also interesting contrasts between evidence and usual practice informed by experience. Through continual iterative review and analysis, the final algorithm was finally presented in a format which described management of the three infections in terms of initial assessment of the resident, observation of the resident and action by the care home staff.

**Conclusions:**

This study has resulted in an updated algorithm targeting key infections in care home residents which should be considered for implementation into everyday practice.

## Background

Many older people live in care homes (with or without nursing), the term used in the United Kingdom (UK) for facilities providing services for older people. There are serious concerns about antimicrobial, particularly antibiotic, prescribing in this population [1, 2]. Apart from important implications for individual residents, there are broader implications for the development of antimicrobial resistance (AMR).

Previous research found that from 21 European countries/jurisdictions, Northern Ireland (NI) care homes with nursing had the highest levels of, and greatest variation in, antimicrobial prescribing, with England ranked fourth [3]. Similar findings were reported for residential homes (facilities which are not required to have qualified nursing staff) [4]. Several UK policy documents have emphasised the importance of better stewardship of antimicrobials, and the subsequent minimisation of resistance at both patient and community levels [5–7].

A 2005 Canadian study found that a multi-faceted intervention, including use of diagnostic and treatment algorithms and small group interactive training, reduced prescribing for urinary tract infections (UTIs) for care home residents [8]. It is not known if this type of approach is effective in other infections i.e. other than UTIs and different health care contexts i.e. beyond Canada. In this paper, we describe the refinement, updating and expansion of an algorithm to assist care home staff to manage residents with suspected infection, thereby building and expanding on the original study [8].

Care home staff are central to decision-making in the prescribing of antibiotics, with general (family) practitioners often accepting staff’s assessment and prescribing antibiotics remotely by telephone [9]. Care staff can also be under pressure from relatives or residents to contact the general practitioner (GP) if there are concerns that a resident may have an infection. The aim of the algorithm is therefore to help care home staff recognise and respond to a suspected infection based on best evidence; giving staff the confidence to know when it is appropriate to call the GP, potentially reducing inappropriate prescribing.

## Methods

We used a multi-faceted process involving a literature review, consensus meeting and focus groups and interviews, alongside continual iterative internal review and analysis within the research team to refine and update the algorithm.

### Literature review

We did a rapid scoping review of the literature to obtain the most up-to-date evidence for the management and diagnosis of the most common infections in older people living in care homes, namely, UTIs, respiratory tract infections (RTIs) and skin and soft tissue infections (SSTIs) [10]. We considered systematic reviews, guidelines, reports, review articles and clinical trials published between 2000 and 2016.

#### Search methods for identification of studies

We searched the following electronic databases: The Cochrane Central Register of Controlled Trials (*The Cochrane Library* 2016, Issue 4), MEDLINE Ovid (1946 to May Week 12,016), EMBASE Ovid (1980 to 2016 Week 41), CINAHL plus EBSCO (1980-May 2016) PubMed (1996 – May 2016), and SCOPUS (1983 – May 2016), supplemented with forward citation tracking. We contacted experts in the field of antimicrobial and geriatric medicine for advice on further potential studies. We conducted a ‘grey’ literature search of the National Institute for Health and Care Excellence (NICE), the European Centre for Disease Protection and Control (ECDC), the Infectious Disease Society of America (IDSA), the Society for Healthcare Epidemiology of America (SHEA), and the National Health Service (NHS).

#### Screening, data extraction and management

All titles and abstracts retrieved by electronic searching were downloaded to a reference management database and duplicates removed. Two review authors (AC, CS) independently examined the remaining references and assessed the eligibility of full text papers. Quality assessment of publications was not done as a single appraisal approach was not possible with the range of publications types. Three authors (AC, DE, CS) independently reviewed and extracted data from relevant publications, discrepancies were resolved by an additional author (CH).

### Consensus meeting

#### Consensus approach

We convened a consensus meeting with a range of stakeholders (ethical approval obtained from the School of Pharmacy Ethics Committee 019PMY2016 and all participants provided written, informed consent) with clinical expertise. These participants were recruited through personal contacts of the research team. Before the meeting, each participant was provided with a copy of a draft algorithm, alongside updated evidence on the key target infections.

**Fig. 1:**
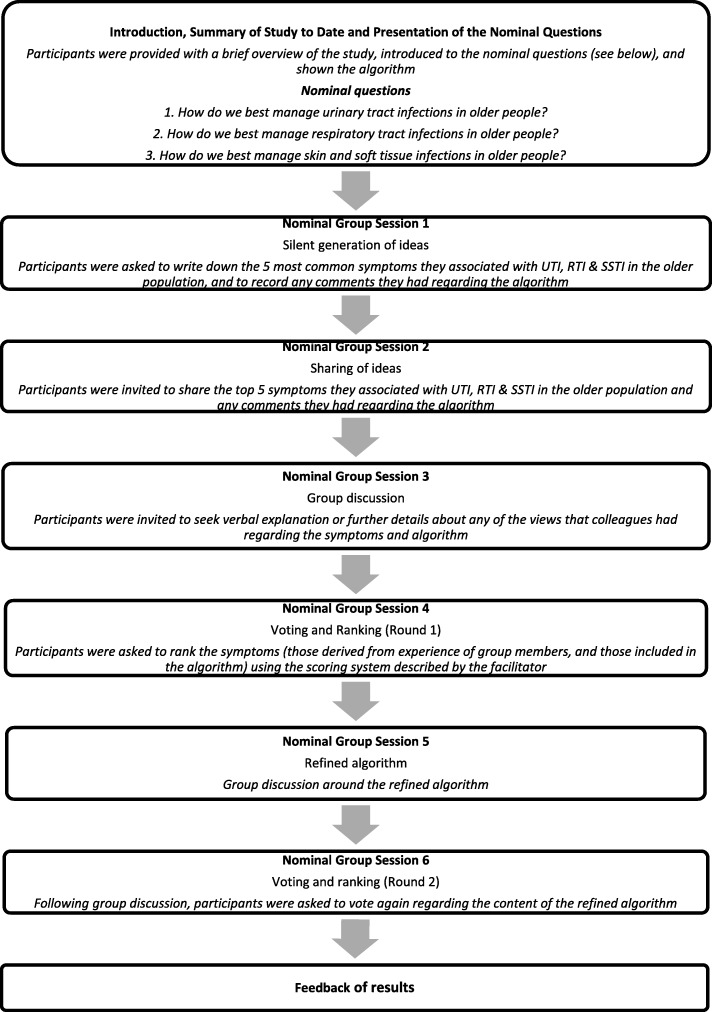
Outline of the consensus meeting

The consensus meeting format was based around the nominal group technique (NGT) [11]. Three nominal questions were formulated as presented in Fig. 1. Initially, each participant recorded their views regarding the nominal questions independently. Participants then shared their views and a facilitator led a group discussion. Individuals then voted privately on the suggestions and the results were presented to the group in aggregate. Following further discussion, a final vote determined the signs and symptoms of each infection to be included in the resulting draft of the algorithm.

### Focus groups and semi-structured interviews

We conducted focus group interviews with care home staff (Additional file 1) and relatives of residents (Additional file 2) in six care homes recruited to participate in a future feasibility study and purposively sampled to include three in NI and three in West Midlands (England) with two nursing homes and one residential home in each area. We also conducted semi-structured one-to-one interviews with GPs (Additional file 3) associated with these homes (ethical approval for focus groups and interviews from the Office for Research Ethics Committees Northern Ireland 16/NI/0003; written informed consent obtained). Topic guide questions were developed using three constructs of the Normalisation Process Theory (NPT); coherence, cognitive participation, and collective action as a means to explore current practice and use of the algorithm as outlined in Table 1 [12, 13].

**Table 1:** Application of three constructs of the normalisation process theory in topic guides for focus groups and semi-structured interviews

• Making sense (coherence): How do participants understand the issue of antimicrobial resistance and what is their usual practice?	
• Engagement and commitment (cognitive participation): What do participants see as necessary to engage staff in the new practice (use of algorithm)?	
• Facilitating the use of the intervention (collective action): How do participants envisage the intervention working and what are the factors which may facilitate or inhibit its use?	

All focus groups/interviews were digitally recorded, transcribed verbatim, and transcripts anonymized. Data analysis was based on the constant comparative method. A selection of focus group and interview transcripts were first open coded inductively, with codes created from patterns in the data. This generated an initial thematic coding frame which was then applied to subsequent transcripts and iteratively refined as new codes were defined. We used the framework matrix facility within NVivo® to assist the analytic process.

### Updating and adaptation of the algorithm

Internal review within the research team was an iterative process throughout all stages relating to the updating and adaptation of the algorithm. Monthly meetings were held involving all members of the research team and each draft of the algorithm were discussed and debated. Changes were made based on results from the literature review, consensus meeting, focus groups and interviews. The final algorithm was agreed upon by all members of the research team.

## Results

### Literature review

**Fig. 2:**
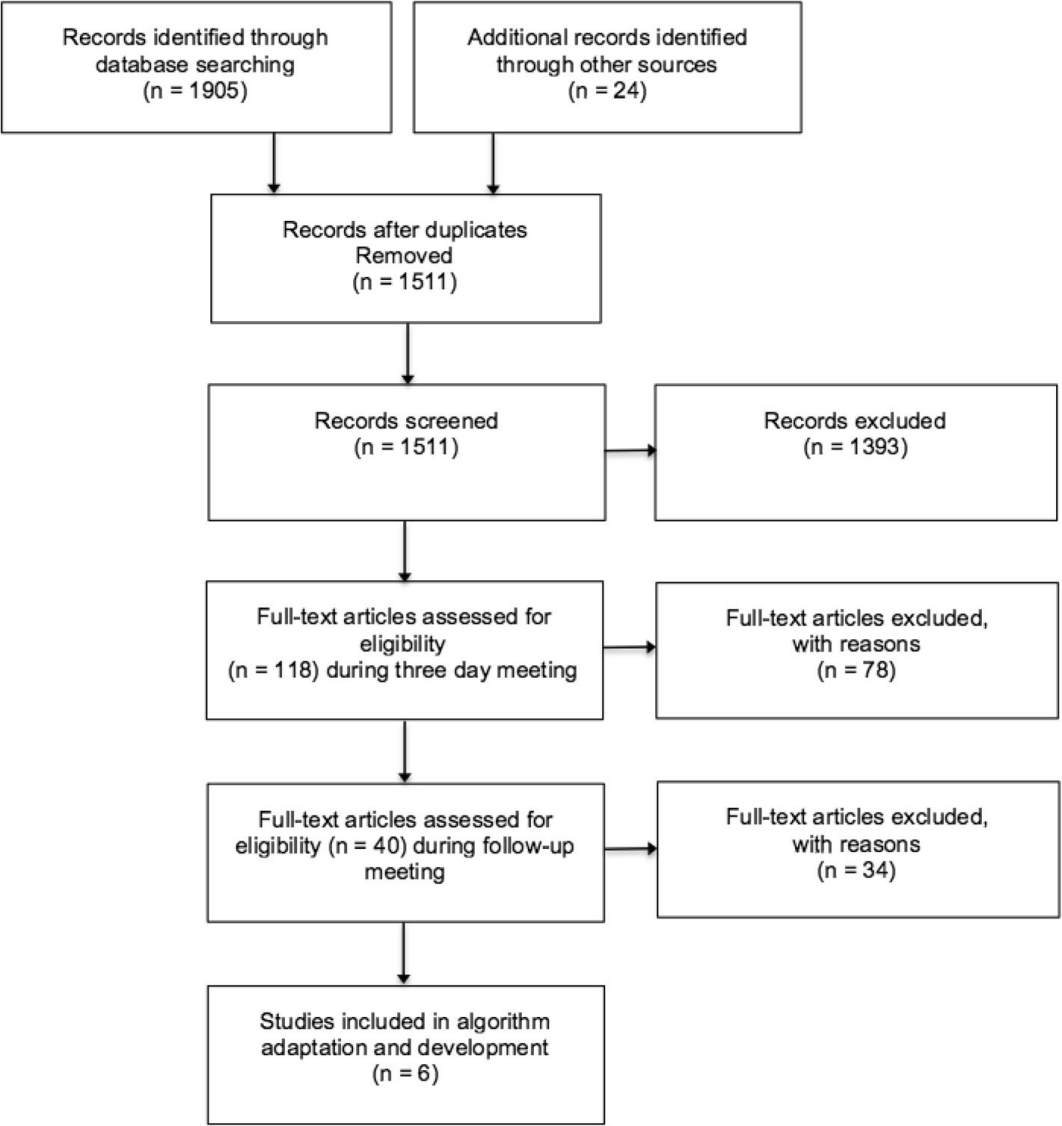
PRISMA diagram outlining the review process for identification of new evidence

An overview of screening and assessment of all papers/resources is summarised in Fig. 2. Forty full-text articles were initially considered eligible; following a full review, a further 34 articles were excluded because they did not provide any updated evidence in relation to UTI, RTI or SSTI. The extracted evidence from the remaining six papers [14–19] is summarised in Table 2.

**Table 2:** Data extracted from the articles contributing to updating of the algorithm

Author & Year	Setting	Design	Population & Condition	Objective	Relevant information for updating/refining algorithm	Group agreement for updating algorithm
Falcone et al. [14]	Community and hospital (includes care home setting)	Narrative review	Older people, Pneumonia [Community Acquired Pneumonia (CAP), Healthcare Associated Pneumonia (HCAP) and Hospital Acquired Pneumonia (HAP)]	This review sought to produce a summary of therapeutic recommendations on the basis of the most up to date clinical and pharmacological data.	Signs and symptoms most commonly associated with pneumonia: cough, fever, chills, pleuritic chest pain. Extra-pulmonary symptoms such as nausea, vomiting, alternation to sensory stimuli or diarrhoea may also be present. It is important to remember that pneumonia in older patients tends to occur more often with extra-pulmonary manifestations. For example, the appearance of a delirium or acute confusion is found in approximately 45% of elderly patients with pneumonia.	Agreed to add in extra-pulmonary symptoms.
Juthani-Mehta et al. [15]	Nursing home	Prospective observational cohort study	Older people, UTI	To identify, among non-catheterised nursing home residents with clinically suspected UTI, clinical features associated with bacteriuria plus pyuria.	The most commonly reported clinical features for suspected UTI in this cohort were change in mental status (39%), change in behaviour (19%), change in character of the urine (i.e., gross haematuria and change in the colour or odour of urine; 15.5%), fever or chills (12.8%) and change in gait or a fall (8.8%). Dysuria, change in character of urine, and change in mental status were significantly associated with the combined outcome of bacteriuria plus pyuria. Absence of these clinical features identified residents at low risk of having bacteriuria plus pyuria (25%), while presence of dysuria plus one or both of the other clinical features identified residents at high risk of having bacteriuria plus pyuria (63%).	Change in character of urine (i.e., gross haematuria and change in the colour or odour of urine) was considered but not supported by more recent guidelines.
Scottish Inter-collegiate Guidelines Network (SIGN) 88, [16]	All settings	Clinical Practice Guideline	Older people, UTI	To provide guidance in the diagnosis and management of suspected UTI in older people	Algorithm to be used in people/residents with fever defined as temperature > 37.9 °C or 1.5 °C above baseline. First stage of algorithm used to differentiate between UTI, RTI, SSTI and gastro-intestinal infection. Advises against using dipstick test in diagnosis of infection. Provides supportive care advice.	Agreed to add supportive care advice to algorithm.
Stone et al.– updated McGeer [17]	Long-term care	Position paper	Older people, infection (general)	To update the 1991 McGeer criteria (Infection surveillance definitions for long-term care facilities) using an evidence-based structured review of the literature in addition to consensus opinions from industry leaders including infectious diseases physicians and epidemiologists, infection control specialists, geriatricians, and public health officials.	Acute swelling of the testes, epididymis and prostate should be included in surveillance definitions for UTIs as these symptoms are a common complication of UTI in both catheterised and non-catheterised males	Agreed to add acute swelling of testes, epididymis and prostate.
D’Agata et al. [18]	Nursing home	Prospective study	Older people, UTI	To describe the presentation of suspected UTI in nursing home residents with advanced dementia and how they align with minimum criteria to justify antimicrobial treatment.	In long-term care residents with dementia, the most common reason for suspected UTI was a change in mental status (44.3%).	Agreed to sub-divide the UTI element of the algorithm into two sections to account for two populations within care homes - those with and without dementia; changed in later iterations
Rowe et al. [19]	Nursing home	Review	Older people, UTI	This review sought to provide an overview of the prevalence, diagnosis and diagnostic challenges, management, and prevention of UTI and asymptomatic bacteriuria in older adults.	The most commonly reported clinical features for suspected UTI in this cohort were change in mental status (39%), change in behaviour (19%), change in character of the urine (i.e., gross haematuria and change in the colour or odour of urine; 15.5%), fever or chills (12.8%) and change in gait or a fall (8.8%) [Juthani-Mehta et al., 2009 – see above].	Change in character of urine (i.e., gross haematuria and change in the colour or odour of urine) was considered but not supported by UK SIGN guidelines.

This evidence was used to develop and update three algorithms, one for each infection of interest. The UTI algorithm was developed starting with the original algorithm by Loeb et al. [8], and updated with the new evidence identified during the literature review (Table 2). The RTI decision-making algorithm largely mirrored that of Loeb et al. [20] with the addition of extra-pulmonary symptoms as per Falcone et al. [14]. Because no new evidence was identified from the literature regarding SSTI, it was agreed that the minimum criteria for initiation of antibiotic therapy for suspected SSTI in long-term care facilities by Loeb et al. [20] should be used. In all three infections, a supportive care step (monitoring, pain relief and fluids) was added as per SIGN 88 guidelines [16].

### Consensus meeting

The consensus meeting took place at the School of Pharmacy, Queen’s University Belfast on 12th September 2016 and consisted of a hospital consultant (respiratory medicine) with experience of prescribing in older people, a GP, a geriatrician and an expert in microbiology. The participants viewed the presented algorithm positively and felt that it had a place within the care home setting, particularly where there was nursing support. However, concerns were expressed about relying solely on temperature, the application of the algorithm in residents who had dementia and how non-nursing staff would use it.

Two rounds of ranking of symptoms by participants took place, with refinements of the algorithm (Fig. 1) being made, with urgency, frequency and incontinence being prefixed by ‘new or worsening’*,* and replacing the term ‘dyspnoea’ with ‘difficulty breathing’, to ensure understanding by all staff.

### Focus groups and semi-structured interviews

Twelve focus groups were conducted during September–October 2016 in NI and West Midlands (England): six with care home staff [one group in each home (participant range 4–9; total = 41)] and six with families of residents (one per home, participant range 4–8; total 28). Semi-structured one-to-one interviews were conducted with eight GPs during January–March 2017 (five in NI and three in West Midlands).

The themes generated from the analysis were structured according to three key aspects of the algorithm: Initial assessment of the resident; Observation of the resident; and Action by care home staff.

#### Initial assessment of the resident

Care home staff described many examples of new or worsening non-specific symptoms that they thought may indicate an infection. Care home staff reported observing change in behaviour such as reduced mobility, increased confusion, agitation or aggression, poor appetite, lethargy, changes to fluid intake and output, not recognising their relative or just ‘not being right’ (Table 3).

**Table 3:** Summary of quotes from focus groups and semi-structured interviews

**Identifiers**: A -F represents each participating home; ‘Staff’ and ‘Family’ represents data from the focus groups and ‘GP’ represents data from a GP interview.	
**Initial assessment of the resident**	
*Well they’d still be, you’d be going by their mobility, because they’d be off their feet, they’d be shaky, they’d be clammy confused, you know a lot of those symptoms. (…) We see a lot of changes in mobility or increased falls when somebody has an infection.* (C: Staff)	
*They manifest like agitation, anxiety or different from what they are. For example, they are always pleasant to the staff and other patients and their families, they can be different when they come in and then the staff go ‘there’s something wrong, something’s not right’.* (A: Staff)	
**Observation of the resident**	
*I think I would be worried about the one to 4 h I would worry about the time, if the answer is no, I think it should be a shorter period of time. (…) Usually an increase in temperature is a good sign that there’s something going on?* (A: Family)	
*You see there, the over- 65 with COPD and delirium is much more clinically urgent than someone with increased frequency of their urine. And yet the end result of that algorithm is phone GP. Now if you phoned the out of hours’ service at three o’clock in the morning* [for COPD resident] *that’s reasonable. If you call the out of hours’ service at three o’clock in the morning like that* [for resident with increased frequency]*, that’s unreasonable* (D: GP)	
*See where it says take the resident’s temperature we obviously can’t do that, step one. (…) That’s why we rely wholly on behaviour and that because we don’t have a lot of tools that we are allowed to use. (…) We would love to be able to take temperatures and things like that there but (*Name of care home*) frowns upon it.* (C: Staff)	
Additional signs and symptoms	
*We would notice a difference in their mood or the way they are, or confusion would be a big thing with elderly people (…) temperature would nearly be the last thing I would take. I would look at all the other things first and then I would take temperature, the GPs will always ask for the temperature.* (B: Staff)	
Urinary tract infections	
*Yeah and if someone is incontinent you don’t always know about the urgency of it because with dementia, not everyone can tell you when you need to go so it makes it quite difficult. And with the lower abdominal pain not everyone will tell you if they are in pain* (F: Staff)	
*I don’t think anyone ever tells us that they’ve got that it’s burning. Because a lot of them are already incontinent, they are wearing incontinent pads, you’re not going to see the increased urgency or frequency or increased incontinence, so it’s rare that we actually see blood in the pad when they’ve had a urinary infection. A lot of them can’t tell you if they’ve got a lower abdominal pain okay, you could see the shaking and the rigors but that’s not a symptoms that we see often.* (D: Staff)	
Respiratory tract infections	
*See it’s not mentioning here the sputum, the colour of the sputum because COPD, every patient of COPD has sputum (…) you can see a lot from the colour. When it’s infection it’s yellowish, greenish.* (A: Staff)*.*	
Skin and soft tissue infection	
*If they have pus draining from a wound, we always swab it and send the swab. Always. We would never leave that.* ***(…)*** *And then like one was done the other day and it goes back to the GP, the results and then the GP contacts us and then with the antibiotic and then it comes from the pharmacy, so we always swab a pussy wound.* (B: Staff)	
*If we get someone with an abscess and we let the, once you let the pus out you don’t usually have to give the antibiotic cover, so it’s more the antibiotics needed here rather than whenever there actually is pus draining.* (B: GP)	
*Well, if there’s a wound, we would do that if it’s localised redness heat or swelling or anything like that where there’s no abrasion or no wound then we go through the GP – if there’s a sore or the skin like moisture lesions we see. Or if it looks like breaking, then we go to through the district nurses.* (F: Staff)	
**Action by care home staff**	
*If they are not taking the paracetamol and not taking a drink, I’d be more inclined to contact the GP, because I know I will not get that down, if I can get that down with paracetamol and lots of drinks I would wait, usually.* (B: Staff)	

Some care home participants thought the decision-making algorithm should distinguish changes in behaviour for those with and without dementia. However, the study team felt that it would be difficult to provide a comprehensive list of all examples of change in behaviour as the algorithm would become illegible. Therefore, the examples already provided would remain unchanged, but additional participant suggestions would be included as part of an accompanying training package which was developed to support the implementation of the algorithm. Details of the training package to accompany the use the algorithm have been described elsewhere [21].

#### Observation of the resident

##### Assessing a resident’s temperature

Some care home staff considered 37.9 °C too high a threshold and 4 h too long before contacting the GP (Table 3). However, without exception, staff used temperature to alert them to a possible infection in a resident, in combination with behaviour change, prior knowledge of the resident, and vital signs, and sought to reduce temperature with supportive care.

Similarly, family members expressed concern about a 4 h wait between temperature observations. They did not consider temperature a sufficient indicator of infection in older people and were concerned that the algorithm focused on this. These views were also reinforced by GPs, along with concerns about specific waiting times before contacting a GP.

Staff and relatives at the residential homes reported that residential home staff were not allowed to measure (‘take’) a resident’s temperature using a thermometer, as it was deemed to be a ‘nursing task’. Participants reported that they would monitor a resident’s temperature by: feeling the head of the resident, if the resident was sweating or flushed, or whether the resident wished to remove clothing to cool down.

Initially, the research team considered changing ‘take resident’s temperature’ to ‘assess resident’s temperature’ to accommodate this practice. However, it agreed that it would be more useful to train staff to use thermometers in the residential homes.

##### Urinary tract infections

Participants expressed concern that it was challenging to assess new or increased urgency, frequency or incontinence, blood in urine and lower abdominal pain in care home residents, particularly in those who are incontinent or have dementia (Table 3). They also noted that they would place more importance on some symptoms, such as evidence of blood in the urine, change in smell or colour of urine and dehydrated skin. However, these were not added to the algorithm, because of lack of supporting evidence.

Participants who were registered nurses described how they would not expect to observe shaking or rigors in residents with a temperature below 37.9 °C. Evidence supported a 1.5 °C increase in baseline temperature for UTIs, which may not necessarily be greater than 37.9 °C, [16, 20], so again, no changes were made.

##### Respiratory tract infection

Nursing home staff described some concerns about the algorithm, for example, that a respiratory rate > 25 would often warrant emergency assistance (ambulance). It was reported that residents with RTIs can deteriorate very quickly and waiting 4 h before contacting GPs could be too long. Staff described how, in their experience, green/yellow sputum may indicate infection (Table 3), however, evidence did not support this inclusion, so no changes were made.

##### Skin and soft tissue infection

Staff from nursing homes reported taking swabs from a wound producing pus and results from the swab test would be sent to the GP who would then prescribe an antibiotic. Conversely, GP indicated that if pus was draining from a wound, an antibiotic was not usually required (Table 3).

Participants also discussed if they would wait until there were two or more of the other symptoms listed in the algorithm for SSTIs before contacting the GP. Residential home staff reported that they would contact a district nurse in the first instance, who would then contact the GP if needed.

#### Action by care home staff

Care home staff participants described how they felt the algorithm generally reflected usual practice. When a resident had a temperature greater than 37.9 °C, staff provided supportive care (e.g. fluids and paracetamol) and re-checked the temperature approximately 2 h before contacting the GP.

### Updating and adaptation of the algorithm

The final algorithm developed through the process described was structured as described below.

#### Initial assessment of the resident

The research team concluded that the most appropriate way to begin the algorithm was provision of a list of non-specific signs and symptoms of infection (including change in behaviour in the resident), followed by specific signs and symptoms of each of the three infections.

#### Observation of the resident

It was agreed temperature should not be used as a stand-alone criterion in the initial assessment of the resident (as was the case in the original algorithm) [8], as older people do not always present in this way when they are unwell [22, 23]. Additionally, an increase of 1.5 °C above baseline (denoted in the original algorithm) [8] was not applicable as baseline temperatures are not routinely recorded in UK care homes. However, assessment of temperature was included in this step to identify residents who may be extremely unwell, with a value of 37.9 °C or more requiring additional monitoring. Following measurement of temperature, this stage also incorporated specific signs and symptoms of each infection.

##### Action by care home staff

An action stage was added to the end to instruct care home staff on how to proceed, depending on presenting symptoms. For example, if the resident fulfilled the criteria for a suspected UTI; i.e. two or more symptoms from the list or dysuria alone, the GP should be contacted. If the minimum number of symptoms were not present, staff were instructed to monitor residents with a temperature between 37.3 °C and 37.9 °C. In this scenario, the staff member was instructed to repeat Step 1 (taking the resident’s temperature) after 6 h.

**Fig. 3:**
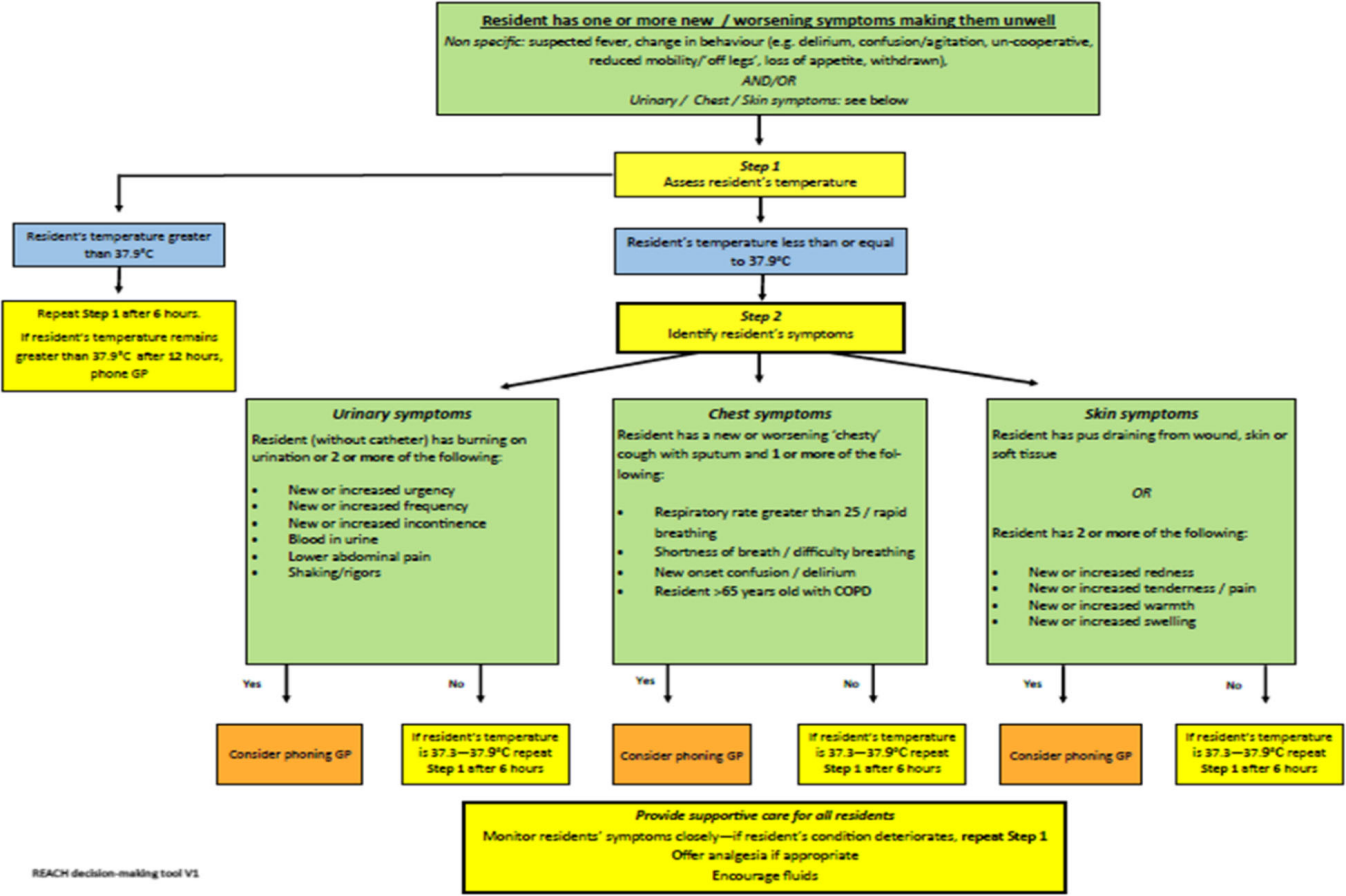
Revised and adapted version of the algorithm

A copy of the final algorithm is shown in Fig. 3.

## Discussion

We set out to adapt, expand and update the decision-making algorithm and training material [21], developed by Loeb et al. [8]. This was achieved through a robust and rigorous approach to updating of all material, whilst navigating through the tension between published evidence and ingrained clinical practice.

The scoping review revealed there were relatively few new publications in managing the three key infections, with the most recent papers being published in 2013 [18, 19]. The included papers largely focused on UTIs, which is the most common infection type in care home residents [9]. This is reflected in the recent publication of two algorithms focusing on the diagnosis and treatment of UTIs in older people [24, 25]. Although there are areas of commonality in these two latter algorithms and the UTI aspects of our work, there are some differences as well. These may be attributed to the different methodological approaches taken, the different contexts (one study was conducted in the USA while this present study was undertaken in the UK) and our consideration of the residential home setting. Our approach was more holistic, involving input from a range of different healthcare professionals, and not just specialist physicians. Indeed, the starting point for the REACH algorithm were non-specific symptoms, rather than a narrow focus on UTI symptoms. Furthermore, urinalysis is under the remit of the GP and not that of the nursing staff in UK care homes. The lack of new research may be due to the relative paucity of research in the care home population, and difficulty in establishing definitive diagnostic criteria for infection in this population. Three papers referred to change in mental status as being important in UTI identification [15, 18, 19]; however, this is difficult in a population in which cognitive impairment is common. There was discussion as to whether two management ‘pathways’ should be presented for UTIs; i.e. one for those with dementia, and those without, along with separate algorithms for each infection. However, it was agreed that a combined algorithm, with a common starting point, followed by three ‘pathways’ for each of the infections would be the preferred form of presentation.

The consensus approach facilitated a consideration of the key symptoms. Temperature was identified as important, but interpretation was seen as problematic as a rise in temperature was not always indicative of infection [22, 23]. Confusion and cognitive impairment were recognised as difficult in residents (confirmed by the literature review), and the consensus meeting participants recommended staff should be aware of changes in residents’ behaviour as this may indicate infection rather than confusion per se.

The focus groups and interviews generated rich and complex data. Participants could see the value in the algorithm, and staff often reported that it reflected their usual practice. The findings also reflected the issues that had been raised in the literature review and consensus meeting: the challenges presented by confusion in this population, concerns regarding interpretation of temperature and that staff in residential care homes did not usually measure temperature. There were also interesting contrasts between evidence and usual practice informed by experience. Care home staff frequently reported on the smell of urine as being indicative of infection, but the SIGN guidelines [16] had not included this. Other publications (albeit ones which did not meet the criteria for inclusion in the rapid review) have highlighted this symptom as being problematic in terms of evidence for a UTI [26].

Involving key stakeholders was challenging as it elicited a huge body of data and sometimes contradictory views compared to published evidence. However, it may have engendered a sense of ownership of the algorithm. Goeman et al. [27] showed the value of a co-creation approach in developing a model of care for dementia support.

The scoping review, the consensus exercise, focus groups and interviews contributed to the ongoing discussions and work of the research team who were refining and updating the decision-making algorithm on an iterative basis. This was particularly challenging as data emerging, notably from staff focus groups, reflected practice that was often not supported by evidence. Fossey et al. [28] found that despite a range of evidence-based support materials to promote person-centred care for those with dementia in care homes, many interventions that were being employed did not meet recognised quality standards, and few had been evaluated in trials.

This study has a number of limitations. Although the process which we undertook to develop and adapt the algorithm was extensive and comprehensive, the consensus group was small (*n* = 4). Caveats associated with qualitative work must be considered i.e. findings may not be generalizable, and participants were drawn from a small number of homes and practices. However, reflexivity and standard approaches to data analysis and interpretation were employed. Adapting the algorithm was supported through a number of approaches, including iterative and internal review by the research team which included experts in infection diseases, general practice and pharmacy.

## Conclusion

We have produced a revised and adapted algorithm to encompass the management of the three most common infections in care homes. This has distilled current best evidence and stakeholder experience, into a practical tool suitable for introduction into care homes and should be considered for implementation in everyday practice.

## Supplementary information

**Additional file 1.** Care home staff focus group guide; questions based on knowledge of antimicrobial resistance, usual practice, views on the algorithm, and how data might be collected in the study.

**Additional file 2.** Family members’ focus group guide; questions based on knowledge of antimicrobial resistance, usual practice, views on the algorithm. (DOCX 30 kb)

**Additional file 3.** GP interview guide; questions based on usual practice, views on the algorithm.

## Data Availability

The datasets used and analysed during the course of this study are available from the corresponding author on reasonable request.

## Abbreviations

AMR: Antimicrobial resistance
CAP: Community Acquired Pneumonia
COPD: Chronic Obstructive Pulmonary Disease
ECDC: European Centre for Disease Protection and Control
GP: General Practitioner
HAP: Hospital Acquired Pneumonia
HCAP: Healthcare Associated Pneumonia
IDSA: Infectious Disease Society of America
NGT: Nominal group technique
NHS: National Health Service
NI: Northern Ireland
NICE: National Institute for Health and Care Excellence
NPT: Normalisation Process Theory
PRISMA: Preferred Reporting Items for Systematic Reviews and Meta-Analyses
RTIs: Respiratory tract infections
SHEA: Society for Healthcare Epidemiology of America
SIGN: Scottish Intercollegiate Guideline Network
SSTIs: Skin and soft tissue infections
UK: United Kingdom
UTIs: Urinary tract infections
