# Supplementary material for: Developing evidence-based guidance for assessment of suspected infections in care home residents
Source: BMC Geriatr. 2020 Feb 14;20:59. doi: 10.1186/s12877-020-1467-6 (PMC7023778; doi:10.1186/s12877-020-1467-6)
Supplement: Supplementary file 1 — Additional file 1. Care home staff focus group guide; questions based on knowledge of antimicrobial resistance, usual practice, views on the algorithm, and how data might be collected in the study. [file 12877_2020_1467_MOESM1_ESM.docx]

REACH TOPIC GUIDE: CARE HOME STAFF

# Housekeeping (15 mins)

- Collect consent and arrange for copies to be given to participants (if not already done).
- Get contact details of participants so that payment can be made (if not already done).
- Introduce ground rules for focus group regarding: 1) practicalities (need to record session, role of researcher in guiding discussion but allowing participants to take up the discussion in their own terms, importance of not talking over one another, and encouraging people to voice their opinions); and 2) ethical considerations (seeking agreement regarding voicing disagreements in a reasonable way and maintaining confidentiality outside the group).

# Introduction (5 mins)

- Introduce study and give very brief overview of decision-making aid and data collection forms that will be used by staff in the study (mention the training they will receive). Make clear that the decision aid is used to help care home staff decide whether or not to contact the GP when they think a resident might have an infection.
- Check whether participants have any questions before beginning focus group and recording.
- Begin recording

# Questions (approx. 70 mins)

### Knowledge of AMR (5-10 mins)

## What do you know about antimicrobial resistance?

## Prompt (if AMR appears not to be a familiar term)

## ‘Antimicrobial resistance’ may be too much of a technical term, are terms such as ‘superbugs’ or ‘MRSA’ more familiar to you? Tell me more about what you know about these.

### Usual practice (10-15 mins)

## Tell me what usually happens when you think a resident might have an infection. Take me through it step by step. (Stress we’d like to know what actually happens rather than what they think should happen and who and what is involved in the process)

Prompts

1. What symptoms or signs make you think a resident might have an infection?
2. What do you usually do next? (e.g. take temperature (how –oral/ear/rectal), speak to colleague/GP, consult guidelines, order urine culture, dipstick)
3. And then what? Anything else?
4. Tell me about any notes you take when you suspect a resident has an infection. What information do you usually record? How do you do this and where do you keep these notes (e.g. hard copies or electronic)? How do you usually handover these notes?
5. What else is important when you’re concerned about whether a resident might have an infection and be prescribed antibiotics or not? (e.g. your knowledge of the resident, pressure from relatives, the GP, care home staff, manager)

### Decision aid (approx. 30 minutes)

## Think-Pair-Share 1: Using the decision aid

| - Give very brief introduction to decision aid. - Distribute decision aid and have participants think briefly about on own (1-2 min). Ask them to imagine they have a resident with a suspected infection in front of them and to consider how well they think the decision aid would actually work in practice. - Allow participants to form groups of 2-3 people to discuss tool (3-4 min) (ask them to think about at least one question they’d like to ask, or one comment they’d like to make about it). - Re-group and in larger group ask about their question/comment. Then use the following questions to prompt discussion (25 min). |
| --- |

Prompts

1. Who do you think will use this decision aid?
2. Where do you think it will used? What format should we provide it in (e.g. A4 or A3 size laminated poster – where should the decision aid be displayed or kept)?
3. Is the tool easy to follow? In what ways?
4. Are you confident that using the aid will help decision-making around when to contact the GP and what to say to the GP? In what ways will it help?
5. In what particular circumstances do you think it will be easy to use the aid? (E.g. with particular residents, infections, times of day/year, when working with particular staff, with particular GPs, with particular support)? Why is this?
6. What’s missing? What’s not needed? What’s confusing? (E.g. symptoms/signs)?
7. What concerns do you have about using it?
8. In what circumstances do you think the decision aid will be difficult to use or that it might not be used? (E.g. with particular residents, infections, times of day/year, when working with particular staff, with particular GPs, with particular support)? Why is this?
9. Still thinking about using the aid—who, or what, do you think is essential to help make sure that the it will actually work in practice (e.g. training needs, managerial support, support from GPs, residents, families)? Anyone/anything else?
   1. You’ve mentioned x (particular person/need/support raised by participants), tell me more about why x is so important?
   2. What difficulties do you see if you don’t have x? How would you get around these?
10. How different do you think using the aid will be from what you normally do? (e.g. your activities and relationships with others)

#### Is there anything else you would like to say about the decision aid?

### Data collection form (approx. 20 minutes)

## I’ve already asked you about any notes you take when you think a resident might have an infection, but do you usually collect any other information? If so, tell me more about what type of information you collect and how you do that.

## Think-Pair-Share 2: Using the decision aid data collection form

Imagine you have to fill out this form, think about how well this would work in practice.

| - Distribute data collection form and have participants think briefly about on own (1-2 min). - Ask participants to form groups of 2-3 to discuss and share thoughts (3-4 min) - Re-group and use following questions to prompt discussion in larger group |
| --- |

Prompts

1. Who will complete this form? Will more than one person have to contribute to completing it?
2. When and where do you think it will be completed?
3. How will you keep track of this form in the midst of all the other forms you have?
4. Where will it best be kept before and after it is completed?
5. How easy is the form to follow?
6. What particular difficulties do you foresee in using the form? Why is this?
   1. E.g. with particular residents, infections, busy times of day, when working with particular staff)?
7. What changes would you make to make it easier for you to complete or fit in with your usual practice?

## Is there anything else you would like to say about this data collection form?
