# Supplementary material for: Developing evidence-based guidance for assessment of suspected infections in care home residents
Source: BMC Geriatr. 2020 Feb 14;20:59. doi: 10.1186/s12877-020-1467-6 (PMC7023778; doi:10.1186/s12877-020-1467-6)
Supplement: Supplementary file 2 — Additional file 2. Family members’ focus group guide; questions based on knowledge of antimicrobial resistance, usual practice, views on the algorithm. (DOCX 30 kb) [file 12877_2020_1467_MOESM2_ESM.docx]

REACH TOPIC GUIDE: FAMILY

# Housekeeping (15 mins)

- Collect consent and arrange for copies to be given to participants (if not already done).
- Get contact details of participants so that payment can be made (if not already done).
- Introduce ground rules for focus group regarding: 1) practicalities (need to record session, role of researcher in guiding discussion but allowing participants to take up the discussion in their own terms, importance of not talking over one another, and encouraging people to voice their opinions); and 2) ethical considerations (seeking agreement regarding voicing disagreements in a reasonable way and maintaining confidentiality outside the group).

# Introduction (5 mins)

- Introduce study background, making clear that the decision aid is used to help care home staff decide whether or not to contact the GP when they think a resident might have an infection.
- Check whether participants have any questions before beginning focus group and recording.
- Begin recording

# Questions (70 mins)

## Knowledge of AMR (10 mins)

1. What do you know about antimicrobial resistance?

Prompt (if AMR appears not to be a familiar term)

‘Antimicrobial resistance’ may be too much of a technical term, are terms such as ‘superbugs’ or ‘MRSA’ more familiar to you? Tell me more about what you know about these.

#### I’d like to show you a short video which talks about the massive concern about the high level of prescribing of antibiotics, it particularly focuses on the risks to the general population (show first 1.02 minutes of video [https://youtu.be/7PhmyNBWGik](https://youtu.be/7PhmyNBWGik%20)).

#### The video gives you an idea of the public health risks from AMR but there are also risks to individuals and these are particularly important for older people living in care homes. Over the past few decades several studies have shown that there is substantial use of antibiotics in care homes but that only between 49-62% is prescribed appropriately. This includes prescriptions for residents who are thought to have a UTI or RTI but who don’t. Older people who are treated with antibiotics are at increased risk of adverse reactions including, for example, Clostridium difficile associated diarrhoea^[[1]](#endnote-1)^. What are your views about that?

## Usual practice (10 mins)

1. Thinking about your relatives here at the home, what usually happens when you or the care home staff think they might have an infection?

Prompts

- How do you become aware that they might have an infection, e.g. do the care home staff talk to you about a possible infection in your relative, or do you feel you can talk to staff if you think your relative has an infection?
- What actions do you expect staff to take if your relative has a potential infection?
- What usually happens next? And then what?

1. What are you usually concerned about when you discover your relative might have an infection?

Prompts

- - What concerns do you usually have if your relative is not prescribed an antibiotic?

## Decision aid (approx. 30 mins)

### Think-Pair-Share: The decision aid

| - Give very brief introduction to decision-making aid. - Distribute decision-making aid and have participants think briefly about on own (1-2 min). - Allow participants to form groups of 2-3 people to discuss tool (3-4 min) (ask them to think about at least one question they’d like to ask about it, or one comment they’d like to make). - Re-group and in larger group ask each small group about their question/comment. Then use the following questions to prompt discussion (25 min). |
| --- |

1. What do you think about care home staff using this aid when making decisions about infections in the care home?
2. How different do you think using the aid will be from what staff normally do?
3. What concerns do you have about care home staff using this aid?

#### Prompt

- - What concerns do you have if your relative is not prescribed an antibiotic by the GP as a result of care home staff using this aid?

1. What do we need to do to deal with your concerns?
2. Finally, and before we bring the discussion to a close, is there anything else you would like to say about the decision-making aid?

1. van Buul, L. W., van der Steen, J. T., Veenhuizen, R. B., Achterberg, W. P., Schellevis, F. G., Essink, R. T., ... & Hertogh, C. M. (2012). Antibiotic use and resistance in long term care facilities. *Journal of the American Medical Directors Association*, *13*(6), 568-e1. [↑](#endnote-ref-1)
