# Supplementary material for: Developing evidence-based guidance for assessment of suspected infections in care home residents
Source: BMC Geriatr. 2020 Feb 14;20:59. doi: 10.1186/s12877-020-1467-6 (PMC7023778; doi:10.1186/s12877-020-1467-6)
Supplement: Supplementary file 3 — Additional file 3. GP interview guide; questions based on usual practice, views on the algorithm. [file 12877_2020_1467_MOESM3_ESM.docx]

REACH TOPIC GUIDE: GPs

## Usual Practice

1. How do you usually find out about a resident of a care home who may have an infection?
2. How do you think care home staff currently assess whether a resident has an infection that needs an antibiotic? (Prompt: What signs or symptoms do they prioritise? (E.g. temperature, behaviour change etc.) What signs or symptoms would you like them to prioritise?)
3. How do care home staff usually convey their assessment of the resident to you? (Prompt: Do they use any particular method of communicating this assessment to you e.g. SBAR? [Situation: Background: Assessment: Recommendation])
4. What influence do care home staff or family members have on any decision you might make regarding prescribing antibiotics for residents of care homes?

1. What else influences any decision you make regarding prescribing antibiotics for residents of care homes? (Prompt: knowledge of the resident and their history of infection; whether you are able to make a visit to the resident).

## Using the decision aid (show aid and let think briefly about it on own)

1. Imagine the care home staff have a resident with a suspected infection. How do you think the decision aid will actually work in practice?
2. How easy or difficult is it to follow?
3. In which ways do you think using the decision-aid will help staff decide when to contact the GP?
4. In which ways do you think using the decision-aid will help staff convey their assessment of the resident to you?
5. Which staff do you think would use the aid? (E.g. nurses, senior care assistants, junior care assistants).
6. Are there particular circumstances in which you think it will be easier or more difficult to use the tool than others? (E.g. with particular residents, infections, times of day/year)? Why?
7. Do you foresee any problems in using this tool during an outbreak of a respiratory virus? What changes could we make to the aid to prevent this?
8. Is there anything missing? (Prompt: symptoms/signs. Anything confusing?)
9. What concerns do you have about care home staff using it?
10. How do you think using the decision-aid will change how care home staff usually assess whether a resident has an infection that needs an antibiotic?
11. Is there anything else you would like to say about the decision-making tool?
